# Catalytic activity of a novel serine/threonine protein phosphatase PP5 from *Leishmania major*

**DOI:** 10.1051/parasite/2014027

**Published:** 2014-06-04

**Authors:** Brianna Norris-Mullins, Paola Vacchina, Miguel A. Morales

**Affiliations:** 1 Department of Biological Sciences, Eck Institute for Global Health, University of Notre Dame Notre Dame IN 46556 USA

**Keywords:** signaling, phosphatases, mutagenesis, activity, drug target

## Abstract

Leishmaniasis is a vector-borne disease caused by protozoan parasites of the genus *Leishmania*. Our knowledge of protein phosphatases (PPs) and their implication in signaling events is very limited. Here we report the expression, characterization and mutagenesis analysis of a novel protein phosphatase 5 (PP5) in *Leishmania major*. Recombinant PP5 is a *bona fide* phosphatase and is enzymatically active. Site-directed mutagenesis revealed auto-inhibitory roles of the N-terminal region. This is a rational first approach to understand the role of PP5 in the biology of the parasite better as well as its potential future applicability to anti-parasitic intervention.

## Abstract (fr)

La leishmaniose est une maladie transmise par un vecteur, due à des parasites protozoaires appartenant au genre *Leishmania*. Notre connaissance des phosphatases de protéines (PPs) et leur implication dans la signalisation est très limitée. Nous rapportons ici l’expression, la caractérisation et les analyses de mutagenèse d’une nouvelle PP5 chez *Leishmania major*. La PP5 recombinante est une phosphatase authentique, enzymatiquement active. La mutagenèse dirigée a identifié les rôles auto-inhibiteurs de la région N-terminale de la PP5. Il s’agit d’une première approche rationnelle vers une meilleure compréhension du rôle de la PP5 dans la biologie du parasite, ainsi que pour une utilisation potentielle dans le cadre d’interventions antiparasitaires.

## Introduction

Leishmaniasis is a vector-borne disease caused by protozoan parasites of the genus *Leishmania* [3]. The disease’s medical implications vary from disfiguring cutaneous leishmaniasis to life-threatening visceral leishmaniasis. Leishmaniasis is considered a neglected tropical disease with more than 12 million people currently infected and approximately 2 million new cases emerging each year, though these statistics are largely underestimated due to misdiagnosis and lacking surveillance systems [2]. Notably, co-infection with the human immunodeficiency virus (HIV/AIDS) is more detrimental to patients exhibiting disease, as the infections target similar immune cells and vastly promote clinical progressions of each other [1]. Though there is no vaccine for leishmaniasis, some treatments are available and are, usually, species-specific. Currently the most common drugs used to treat infection are pentavalent antimony, amphotericin B, and miltefosine. Unfortunately, these drugs are toxic and expensive. As resistance is a common feature of most of them, the urge for newer drugs is imperative [10]. During the infectious cycle, *Leishmania* differentiates from the extracellular flagellated promastigote to the intracellular pathogenic amastigote form. Promastigotes develop in the midgut of sandflies and following infection in humans, differentiate to intracellular amastigotes that multiply inside the macrophage lysosome [14]. This differentiation is triggered by environmental signals, mainly acidic pH and high temperature in the mammalian host [24]. Signal transduction cascades often relay these environmental stimuli through reversible phosphorylation via kinases and phosphatases, ultimately leading to changes in protein activity, interaction and expression profiles [11]. Our knowledge of these signaling molecules in *Leishmania* is scarce despite their essential roles in the biology of the parasite. Mitogen-activated protein kinases (MAPKs) are conserved across virtually all eukaryotic organisms. The importance of the core MAPK pathway has been revealed in *Leishmania mexicana* and deletion analysis of LmxMPK1 and 2 showed that both are essential for survival in the amastigote stage [21, 22]. LmxMPK3 regulates flagellar length [9] and LmxMKK is the MAPKK responsible for its regulation [23]. On the other hand, our understanding of the biology of protein phosphatases in trypanosomatids is poor despite the fact that protein dephosphorylation is largely implicated in critical post-translational modifications [15], differentiation [18, 19], and more recently in parasite drug resistance [4]. Approximately 96%–99% of proteins in eukaryotes are phosphorylated on Serine and Threonine (Ser/Thr) residues. Ser/Thr phosphatases are divided into three families: phosphoprotein phosphatases (PPPs), metallo-dependent protein phosphatases (PPM), and aspartate-based phosphatases with a DxDxT/V motif [17]. Protein phosphatase 5 (PP5) is a putative gene belonging to the PPP family. It has a unique characteristic in that it differs from other phosphatases in the PPP family due to its N-terminal tetratricopeptide repeat (TPR) domains, which are momentous in protein-protein interactions and autoinhibition [5]. In mammalian systems, PP5 TPR domains associate with several proteins that affect signal transduction networks, including the glucocorticoid receptor (GR)-heat shock protein 90 heterocomplex [7]. The catalytic domain of PP5 shares 35%–45% sequence identity with the catalytic domains of other PPP phosphatases, including protein phosphatase 1 (PP1), 2A (PP2A), and 2B/calcineurin. It is less abundant than the above-mentioned PPPs in mammalian systems and its basal activity is low under typical phosphatase assay conditions [8]. Here we depict the catalytic activity of a novel protein phosphatase PP5 in *L. major* and dissect the residues implicated in its autoinhibitory regulation.

## Materials and methods

### Bioinformatics

*Leishmania major* PP5 was used as an initial query for PSI-BLAST and sequences corresponding to putative PP5 proteins from the sequenced genomes of *Leishmania infantum, L. braziliensis, L. mexicana, Trypanosoma cruzi, T. brucei, Homo sapiens, Mus musculus, Arabidopsis thaliana, Bos taurus, Schistosoma mansoni*, and *Saccharomyces cerevisae* were retrieved using the TriTrypDB and UniProt databases: (http://tritrypdb.org/tritrypdb/) and (www.uniprot.org). Sequences were aligned with Clustal X (v2.0) and edited with Jalview [20]. Alignments were converted to MEGA compatible files and fed into the MEGA5.2 software package. A Neighbor-Joining tree was computed with 500 bootstrap replicates.

### Molecular constructs

In order to generate a recombinant PP5 protein, a 1.4 kb region containing PP5 was amplified from genomic DNA of *L. major* FV1 using the primers 5′-ACC CTC GAG ATG GAG GAG TCC GAC CGC-3′ (XhoI), R 5′-GCC GCG GCC GCT TAA AAT AGA CCC GCG CC-3′ (NotI) and LongAmp high-fidelity *Taq*-DNA polymerase (New England Biolabs) following the manufacturer’s recommendation. The product was cloned into pGEM-T (Promega) to create pGEM-T-PP5. N-terminal GST-PP5 fusions (Glutathione S-Transferase) were obtained by inserting the 1.4 kb PP5 fragment from pGEM-T into the respective site of pGEX-5x. pGEX-5x-PP5 constructs were then transformed in BL-21 *Escherichia coli* (*E. coli*) cells (NEB). Cells transfected with the empty vector, pGEX-5x, were used as mock controls.

### Expression and purification

Cell lines were grown in LB broth and ampicillin (100 ug/mL). At OD = 0.5 the cultures were induced with 0.1 mM isopropyl-1-thio-*β*-D-galactopyranoside (IPTG) for 2 h at room temperature. Cells were harvested and lysed with BugBuster (Novagen). Recombinant proteins were purified with GST-spin columns (GE Healthcare) as per the manufacturers’ protocol. Following elution each culture was dialyzed against 50 mM Tris-HCl (pH 8.0).

### Western blotting

Proteins were revealed using the following primary antibodies: rabbit polyclonal anti-GST (Santa Cruz Biotechnology), rabbit polyclonal anti-PP5 (Abcam) and anti-rabbit HRP-conjugated secondary antibodies (Pierce). Cell extracts were separated in 4%–12% Bis-Tris NuPAGE gels (Life) and electro-blotted onto PVDF membranes (Pierce).

### Site-directed mutagenesis

GST-PP5 mutants were generated using a QuikChange II XL Site-Directed Mutagenesis Kit (Agilent Technologies) as recommended by the manufacturer. Briefly, primers 5′-CTC CCG CGG CAA CGC CGA GGG ACT CTC G-3′ and R 5′-CGA GAG TCC CTC GGC GTT GCC GCG GGA G-3′ were used to create a H276A mutation, primers 5′-TAC CTG AAG CTG GCG CTG CCT GGA GCG-3′ and R 5′-CGC TCC AGG CAG CGC CAG CTT CAG GTA-3′ were used to create an E51A mutation and primers 5′-CGA CCC TGG CTT TGT GGC GGC GTA CTA CCG CAA G-3′ and R 5′-CTT GCG GTA GTA CGC CGC CAC AAA GCC AGG GTC G-3′ were used to create a K72A mutation.

### Biochemical characterization

Activity of *L. major* recombinant PP5 was measured in a phosphatase assay using p-nitrophenyl phosphate (pNPP) (New England Biolabs) as a substrate. Amounts of recombinant protein were quantified using a Bradford assay. As GST alone can alter protein quantification, samples were further compared with known BSA concentrations using densitometry analysis of a SimplyBlue SafeStain (Invitrogen) stained gel. 1 ug of each recombinant sample, diluted in 1× colorimetric assay buffer (20 mM Tris pH 7.5, 5 mM MgCl_2_, 1 mM EGTA, 0.02% *β*-mercaptoethanol and 0.1 mg/mL bovine serum albumin [BSA]), was added to corresponding wells on a 96-well plate and the reaction was initiated by addition of pNPP. After a 45-min incubation at room temperature, 5N NaOH was added to each well to stop the reaction. Absorbance (ABS) was read using a plate reader at 405 nm. Okadaic acid (OA) and arachidonic acid (AA) were purchased from Santa Cruz and Sigma, respectively. Statistical analysis was performed with GraphPad Prism v. 6.04.

## Results and discussion

**Figure 1.**
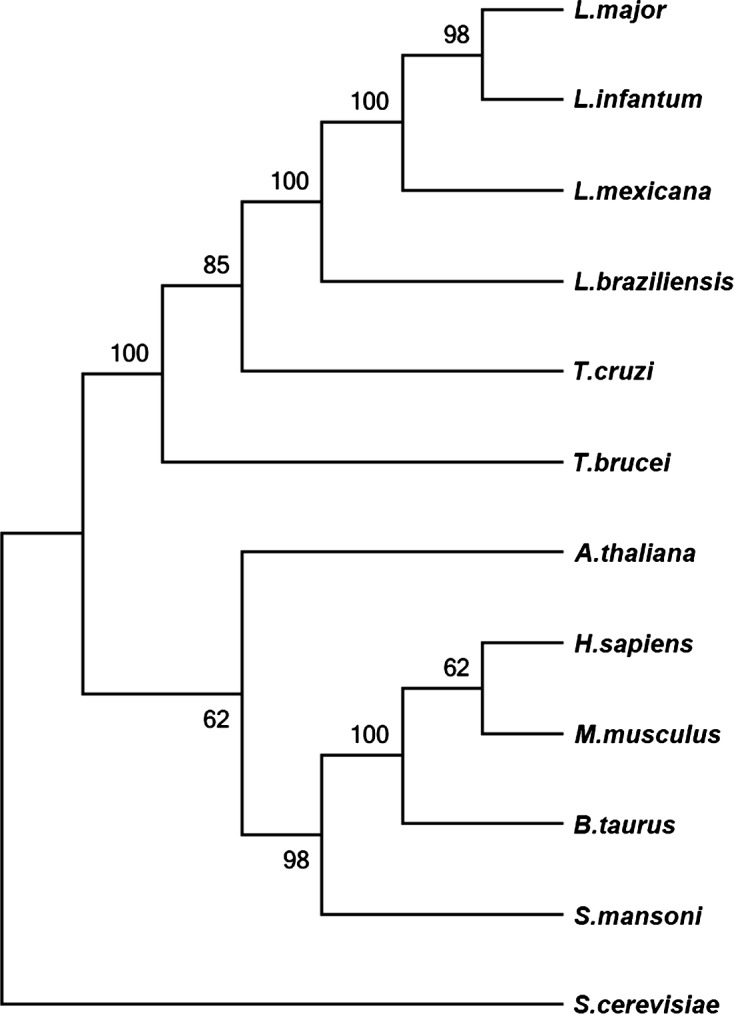
Bioinformatics analysis. The relationship of *Leishmania major* PP5 (XP_001682421.1) to PP5 homologs of *Leishmania infantum* (XP_001464832.1), *Leishmania mexicana* (XP_003874029.1), *Leishmania braziliensis* (XP_001563942.2), *Trypanosoma cruzi* (XP_820611.1), *Trypanosoma brucei* (XP_827850.1), *Arabidopsis thaliana* (NP_565985.1), *Homo sapiens* (NP_006238.1), *Mus musculus* (NP_035285.2), *Bos taurus* (NP_001179178.1), *Schistosoma mansoni* (XP_002580099.1) and *Saccharomyces cerevisiae* (EDN61714.1) was analyzed by multiple alignment and cluster analysis using Clustal X. Alignment was fed into MEGA5.2 software and a Neighbor-Joining tree was computed with 500 bootstrap replicates. Numbers on nodes indicate bootstrap support.

Using in *silico* analysis we interrogated the *Leishmania major* (*L. major*) genome for protein phosphatase 5 (PP5) (XP_001682421). The amino acid sequence of PP5 in *L. major* has a 98% overall identity with PP5 from *Leishmania infantum* (*L. infantum*), 77% with *Trypanosma cruzi* (*T. cruzi*), 70% with *Trypanosoma brucei* (*T. brucei*), 50% with *Arabidopsis thaliana* (*A. thaliana*) and only 46% with *Schistosoma mansoni* (*S. mansoni*), *Mus musculus* (*M. musculus*), and *Homo sapiens* (*H. sapiens*). *L. major* PP5 contains three distinct TPR motifs with lower homology to its mammalian counterparts (Fig. S1). Clustering analysis based on multiple alignment confirmed that *L. major* PP5 is closely related to other trypanosomatid PP5s (Fig. 1).

**Figure 2.**
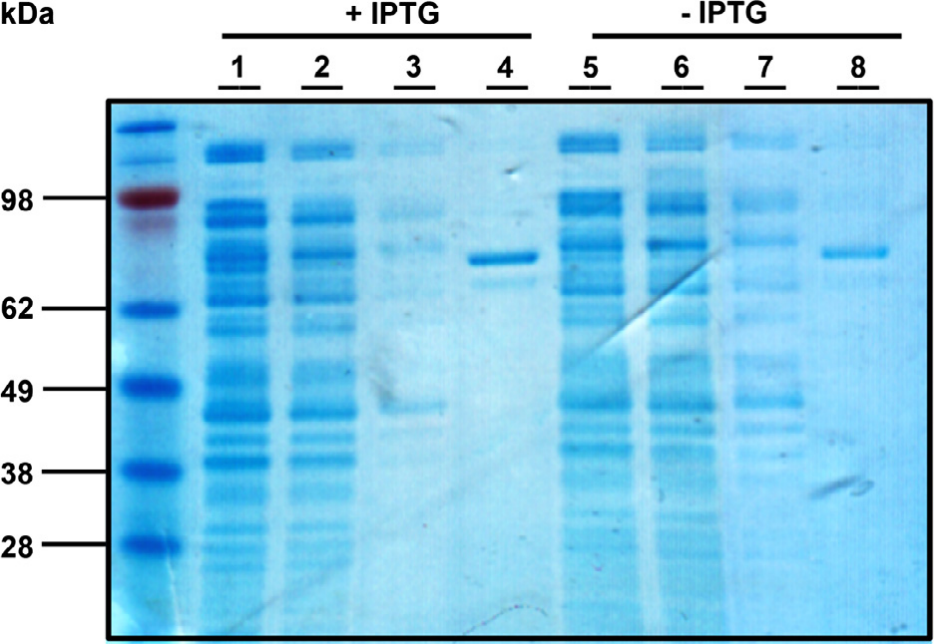
Purification of GST-PP5 from BL-21 cells. Cells were grown to OD = 0.5 and IPTG-induced (0.1 mM) (lanes 1–4) and uninduced (lanes 5–8) cultures were left to stir for two hours at room temperature. Protein was extracted and purified using GST-spin columns. Lanes are representative of the elution process: total protein lysate (lanes 1 and 5), flow through washes (lanes 2–3 and 6–7) and GST-PP5 protein eluate (lanes 4 and 8).

**Figure 3.**
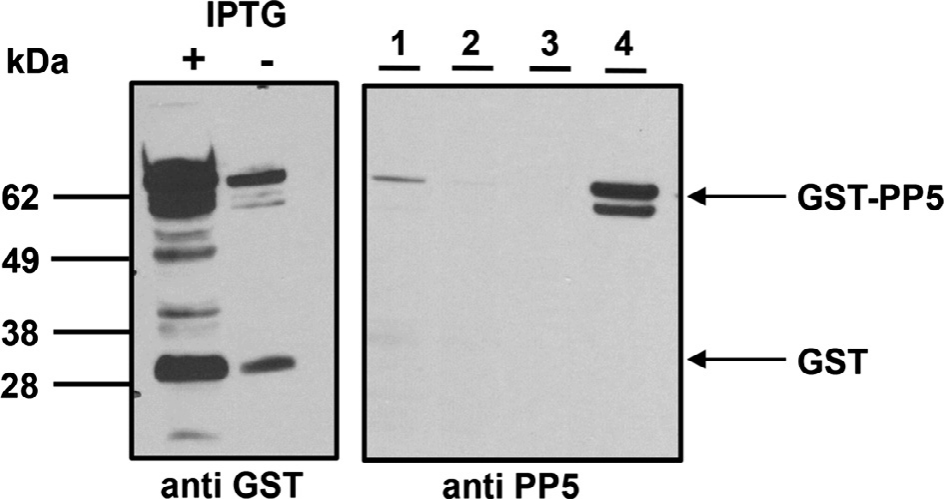
Expression of GST-PP5 in BL-21 cells. Expression of GST-PP5 as detected by Western blotting using an anti-GST antibody (left panel) induced with 0.1 mM IPTG or uninduced. Right panel, polyclonal anti-PP5 antibody. Lanes are representative of the elution process: total protein lysate (lane 1), flow through washes (lanes 2 and 3) and GST-PP5 protein eluate (lane 4). The molecular weight of standard proteins is indicated in kDa.

In order to biochemically characterize PP5 we generated a recombinant GST-PP5 fusion protein which was expressed in BL21 *E. coli* cells. GST-PP5 expression was increased in IPTG-induced cultures as observed by Coomassie staining (Fig. 2, lanes 4 and 8) and Western blot (Fig. 3, left panel) using a rabbit polyclonal anti-GST antibody. To further test the specificity of GST purification, eluted recombinant proteins were separated, electro-blotted onto PVDF membranes and detected with a polyclonal anti-PP5 antibody (Fig. 3, right panel). Prior to performing the activity assay, a gel was run and stained with Coomassie to compare each sample with BSA loading controls via densitometry (Fig. S2-B). This ensured the use of proper amounts of sample for the activity assay, as GST alone could alter protein quantification (Fig. S2-A).

**Figure 4.**
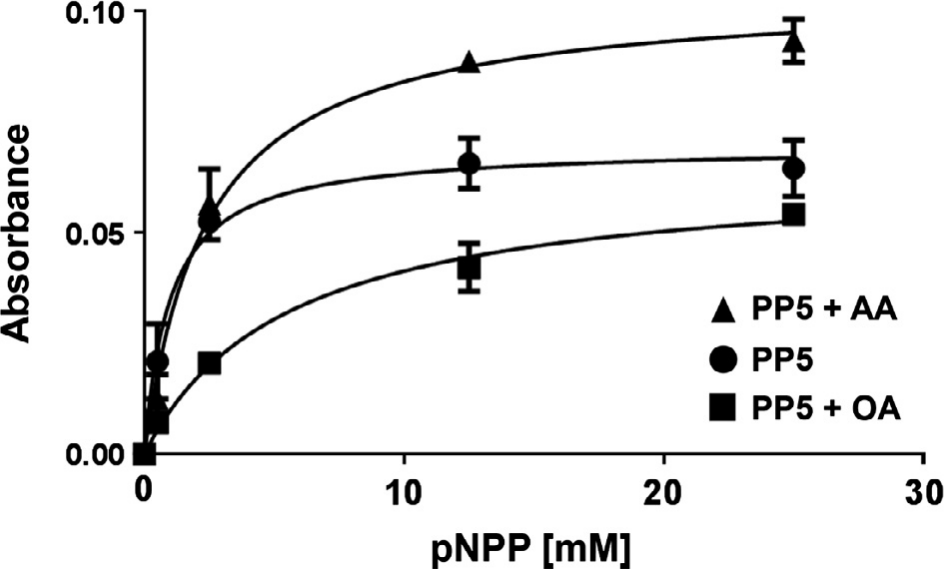
Catalytic activity. PP5 activity was assessed at increasing pNPP substrate concentrations [●] in the presence of 5 μM AA [▲] or 25 μM OA [■] and absorbance was measured on a plate reader at 405 nm. A dose-dependent non-linear regression curve was generated and standard deviation is shown (*n* = 3).

Being the first description of PP5 in *L. major*, we measured the catalytic activity of PP5 using increasing concentrations of p-nitrophenyl phosphate (pNPP) as a substrate. A dose-dependent non-linear regression curve was plotted for the activity of PP5 and PP5 in the presence of 5 μM arachidonic acid (AA) and 25 μM okadaic acid (OA), a generic phosphatase inhibitor (Fig. 4). *L. major* recombinant PP5 is enzymatically active and is inhibited in the presence of OA. Polyunsaturated fatty acids such as AA interact with the TPR domain of PP5, thus activating the enzyme. Our results are in good accordance with the activation of *T. brucei* PP5 in the presence of AA and the regulatory effect of the TPR domain on enzymatic activity [6].

**Figure 5.**
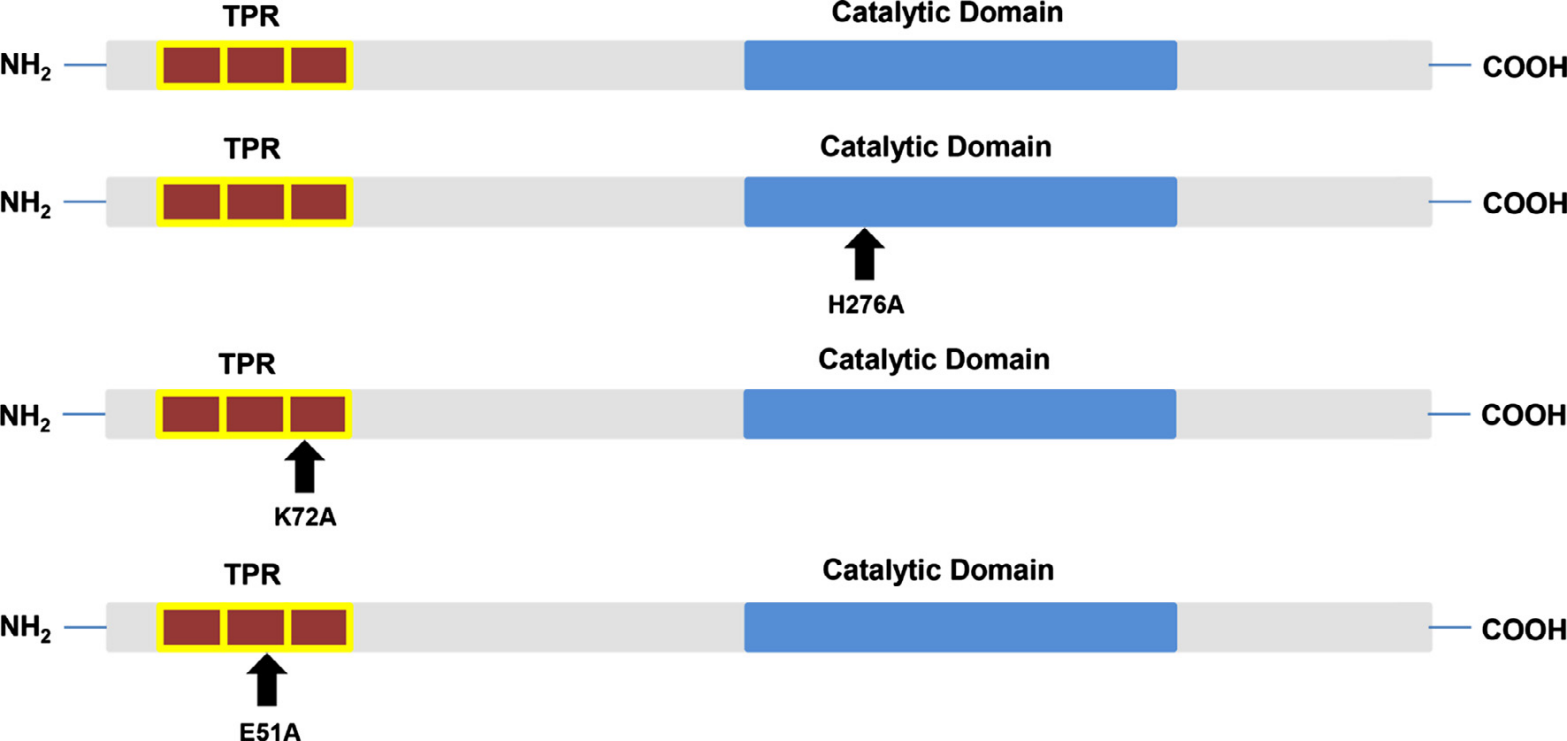
PP5 structure. *Leishmania* PP5 contains a TPR domain made of three TPRs (red) and one phosphatase catalytic domain (blue). Site-directed mutagenesis was performed to change Histidine to Alanine at position 276 [H276A], Lysine to Alanine at position 72 [K72A], and Glutamic Acid to Alanine at position 51 [E51A].

**Figure 6.**
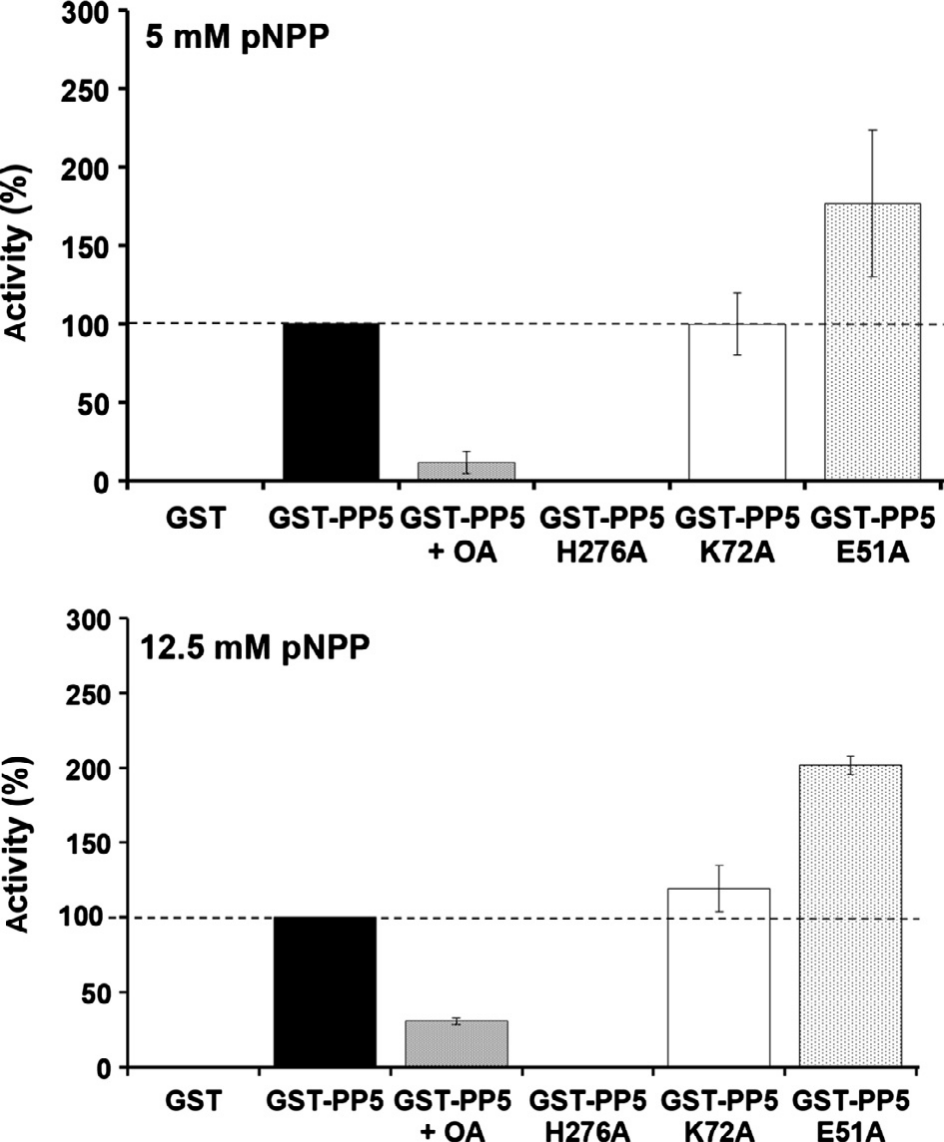
Biochemical characterization of GST-PP5 mutants. Phosphatase activity was assessed at two different pNPP substrate concentrations simultaneously (5 mM and 12.5 mM, respectively) and absorbance was measured on a plate reader at 405 nm. Standard deviation is denoted by the bars shown (*n* = 3).

As mentioned above, the TPR domain on the N-terminal end of PP5 has a known autoinhibitory function [16], therefore we performed site-directed mutagenesis to test the potential negative regulation of a glutamic acid residue (E51A) previously analyzed in mammalian systems [13]. We also mutated a lysine (K72A) residue known to bind to HSP90 [13] and a histidine (H276A) residue known to positively regulate the activity of PP5 [5] (Fig. 5). *L. major* PP5 is active in the presence of 5 mM and 12.5 mM pNPP. 25 μM OA was used as a negative control, although complete inhibition was not observed. Glutathione S-Transferase (GST) was assayed as a negative and background control (Fig. 6). H276A mutation on the C-terminal abolishes PP5 activity in good accordance with its mammalian counterpart [5]. As expected, E51A mutation on the TPR domain results in significantly increased activity, regardless of the different pNPP concentrations assayed, suggesting a potential inhibitory role. K72A mutation, which is part of the HSP90-binding groove in the TPR domain [13], has no regulatory function in the catalytic activity of *L. major* PP5. Together these results suggest that *L. major* PP5 is active and is a *bona fide* phosphatase.

The autoinhibitory function of the TPR domain is conserved in *Leishmania* and we found Glu51 to be implicated in this regulation. On the other hand, Lys72 does not negatively regulate the catalytic activity of PP5; however, further studies using endogenous PP5 parasite extracts are necessary to reveal its implication in the potential interaction with HSP90. Protein binding assays with immunoprecipitated recombinant GST-PP5 and total extracts of *L. major* did not reveal interaction with HSP90 (data not shown). It is important to note that our inability to reveal a PP5- HSP90 interaction may be due to the steric hindrance of GST fused N-terminally to PP5, where the TPR domain is located. Thus, we can only speculate that endogenous *L. major* PP5 may interact with HSP90. Additionally, it has been shown previously in the related *T. brucei* that PP5 interacts with and is required to regulate HSP90 function under stress conditions [12].

## Conclusions

The complete genomes of trypanosomatids (www.trytrip.org) have made possible the screening of putative protein phosphatases. The biochemical characterization of this novel PP5 in *Leishmania major* is a rational first approach to further understand the regulation of signal transduction pathways in this parasite. Additional studies are required to assess substrate specificity and in vivo function. Nonetheless, *L. major* PP5 shows low similarity with its mammalian counterparts, suggesting it may be targeted for therapeutic intervention.
